# Germline masculinization by *Phf7* in *D. melanogaster* requires its evolutionarily novel C-terminus and the *HP1-*family protein HP1D3csd

**DOI:** 10.1038/s41598-021-85560-4

**Published:** 2021-03-18

**Authors:** Shu Yuan Yang

**Affiliations:** 1 Department and Graduate Institute of Biomedical Sciences, College of Medicine, Chang Gung University, Taoyuan, 333 Taiwan; 2 Division of Reproductive Endocrinology and Infertility, Department of Obstetrics and Gynecology, Linkou Chang Gung Memorial Hospital, Taoyuan, 333 Taiwan

**Keywords:** Developmental biology, Genetics

## Abstract

Germ cells in *Drosophila melanogaster* need intrinsic factors along with somatic signals to activate proper sexual programs. A key factor for male germline sex determination is PHD finger protein 7 (Phf7), a histone reader expressed in the male germline that can trigger sex reversal in female germ cells and is also important for efficient spermatogenesis. Here we find that the evolutionarily novel C-terminus in Phf7 is necessary to turn on the complete male program in the early germline of *D. melanogaster*, suggesting that this domain may have been uniquely acquired to regulate sexual differentiation. We further looked for genes regulated by *Phf7* related to sex determination in the embryonic germline by transcriptome profiling of FACS-purified embryonic gonads. One of the genes positively-regulated by Phf7 in the embryonic germline was an *HP1*family member, *Heterochromatin Protein 1D3 chromoshadow domain (HP1D3csd).* We find that this gene is needed for Phf7 to induce male-like development in the female germline, indicating that HP1D3csd is an important factor acting downstream of Phf7 to regulate germline masculinization.

## Introduction

Establishing a correct sexual identity and implementing a sex-specific developmental program is a fundamental cell fate decision to be made for cells that are sexually dimorphic. In the germline, the choice to be female or male is typically determined early during embryogenesis, and subsequent development is closely intertwined with this early decision.

In the germline of *D. melanogaster*, sexual information comes from both the surrounding somatic gonad as well as from intrinsic knowledge of a cell’s sex chromosome composition^1–4^. The sex of the soma and the germline needs to be the same for germ cells to develop normally, and discordance between the two leads to germline atrophy. The signal from the male soma to germline is transmitted via the JAK-STAT pathway while the identity of the female signal is not yet identified^5^.

A few germline factors have been shown to cause germline sex reversal in *D. melanogaster.* In the female germline, Sex lethal (Sxl) is a critical female-determining factor and can induce male germ cells to undergo oogenesis if Sxl is ectopically expressed^1–3,6^. In the male germline, the histone code reader Phf7 (PHD finger protein 7) acts in reverse to how Sxl works: overexpression of Phf7 in the female germline can lead to masculinization^7^. Phf7 contains three PHD domains in its N-terminus and can bind methylated H3K4^7,8^. *Phf7* expression is highly enriched in the male germline; expression of this gene in the male germline starts during mid-embryogenesis and continues to be present in the undifferentiated fraction of germ cells in the developing testis including germline stem cells and spermatogonia^9^.

Interestingly, only amniotes and some insects of Diptera have a *Phf7* gene^10^, and *Drosophila Phf7* genes are unique in that they contain extended C-termini that do not match any known protein domains. Moreover, this portion of *Drosophila* Phf7 appears to be rapidly evolving as homology between more distant *Drosophila* species is already lost. These observations lead one to wonder what the role of the C-terminus of *Drosophila* Phf7 would be.

HP1 proteins is a well-known family of factors that promote heterochromatin propagation and these proteins contain histone-associating chromodomains and chromoshadow domains (CSDs) that can serve as an interconnecting platform with other chromatin-associated factors^11,12^. In *Drosophila* genomes, “half” HP1 proteins exist that have just CSDs, and intriguingly most of them appear to be expressed in a germline-biased fashion^13^. These half-HP1 factors have been suggested to regulate retrotransposon silencing in the germline, but they can potentially regulate additional aspects of germline biology.

Here we examine how different domains of Phf7 help regulate the establishment of the male germline sex, especially regarding the unique C-terminus. Our findings indicate that this novel portion of *D. melanogaster* Phf7 is required for the male-determining function of Phf7 and for proper control of the male germline program. We further looked for factors that are regulated by Phf7 in the embryonic germline and reveal a downstream gene, *HP1D3csd (Heterochromatin Protein 1D3 chromoshadow domain)* which belongs to the family of HP1 proteins, that is positively-regulated by Phf7 in the process of germline masculinization.

## Materials and methods

### Fly stocks and crosses

The flies used in this study are cultured on standard media. The strains include: *w*^*1118*^, *Phf7*^Δ*N2* 7^*, Phf7*^Δ*N18* 7^*, vas-GFP*^14^*, EY03023, UAS-Phf7-FL*^7^*, UAS-Phf7-N*^10^*, UAS-Phf7-C, nos-Gal4*^15^*, tra*^*1*^*, Df(3L)st-j7, HP1D3csd*^*f07323*^*, UAS-HP1D3csd.ORF.3xHA.GW, Df(1)ED7441, Dp(1:Y)B*^*s*^* , FM7a,Dfd-YFP.* Stocks were obtained from the Bloomington Stock Center unless otherwise noted.

Sexing was performed using *Dp(1:Y)B*^*s*^ as a Y-chromosome marker in adults whereas the *FM7a,Dfd-YFP* chromosome was used for sexing embryos by crossing male carriers to unmarked females and performing immunofluorescence to detect YFP expression with an α-GFP antibody. The genetic combination used for generating *tra* mutant flies was *tra*^*1*^*/Df(3L)st-j7.*

### Plasmid construction and S2 cell transfections

*UAS-PHF7.C* was made by cloning the C terminal domain of fruit fly PHF7 protein, amplified by primers (forward: 5′-GAATGCGGCCGCATGGCAGTGCCCGTTGCCG-3′, reverse: 5′-CATAACTAGTCTAATCCTTGCGGCTGGCC-3′), into the pUASpB vector^7^ via *Not*I and *Spe*I sites. The construct was used for transgenesis by integration into the attP2 landing site via ΦC31-based recombination (WellGenetics).

Construction of a fusion protein of Gal4 DNA binding domain (DBD) and Phf7 C-terminus controlled by the ubiquitin promoter was achieved by first cloning the ubiquitin promoter-Gal4 segment into pBKS via *Sac*II and *Apa*I sites. Gal4 was then excised with *Mlu*I and *Nco*I, and a segment containing the Gal4 DBD (forward primer: 5′-TCTGCCCGCAGAATAATCC-3′, reverse primer: 5′-GATTCGGCAACGGGCACTGCCGATACAGTCAACTGTCTTTG-3′) and Phf7 C-terminus (forward primer: 5′-CAAAGACAGTTGACTGTATCGGCAGTGCCCGTTGCCGAATC-3′, reverse primer: 5′-CCAAACGCGTTTACTAATCCTTGCGGCTGGCC-3′) assembled by overlap-extension PCR was inserted into those sites.

For transfections, 5 μg each of two plasmids, either pBKS-Ubi-Gal4 and pUASt-6Xmyc-EGFP (a gift from Lab of Haiwei Pi), or pBKS-Ubi-Gal4DBD/PHF7.C and pUASt-6Xmyc-EGFP were electroporated into 10^7^ S2 cells (250 kV, 950 μFD, infinite resistance). GFP expression was analyzed 48 h after transfection on a FACSAria (BD).

### Immunofluoresence staining, in situ hybridization, and hybridization chain reaction (HCR)

To carry out immunofluorescence staining, gonads dissected in PBS were fixed in 4% paraformaldehyde for 15 min at room temperature and incubated with the appropriate primary antibodies overnight at 4 °C followed by staining with secondary antibodies for at least 2 h at room temperature.

Primary antibodies used were: rabbit-α-Vasa (1:250, Santa Cruz Biotechnology), rat-α-N-cadherin (1:20, DSHB), mouse-α-α-spectrin (1:5, DSHB), mouse-α-Sxl (M18, 1:50, DSHB), mouse-α-Bam (1:25, DSHB), rabbit-α-Phf7 (1:2500^9^). Alexa Flour 488, 694, and 647 secondary antibodies (Thermo Scientific) made in goat or donkey were used at 1:500. Images were taken on APOTOME.2 (Zeiss).

For embryonic in situ hybridization, the probe for *HP1D3csd* was synthesized in vitro using T7 RNA polymerase and a template fragment generated by PCR (Primers: 5′-TGCTAAACGATGGCGGACA-3′; 5′-GAATTAATACGACTCACTATAGGGGGGTGCACATGTTTGATCTCC-3′). Embryo preparation and hybridization was performed as previously described^9^.

Hybridization chain reaction (HCR v3.0, Molecular Instruments) staining of *HP1D3csd* transcripts was performed as recommended by the vendor on embryos. Briefly, embryos were dechorionated for 2 min with 100% bleach, fixed in 4.5% formaldehyde, cleared with xylene substitute, hybridized with 2 pmol of probes overnight at 37 °C, and incubated with 6 pmol of fluorescently-labeled hairpins overnight at room temperature for signal amplification. The embryos were subsequently stained for Vas expression to determine the location of germ cells before confocal imaging (Zeiss).

### RNA sequencing of embryonic gonads

18-22 h embryos that contain the *vas-GFP* transgene either normal or mutant for *Phf7 (Phf7*^Δ*N18*^*)* were homogenized by 7 strokes using the loose pestle in a Dounce homogenizer, filtered twice through 70 μm mesh, centrifuged at 850 g for 2 min to collect gonads^16^. GFP-positive gonads were isolated by FACS-sorting on a FACSAria (BD). RNA was then purified from those gonads using RNA reagent (Bioman). Two biological replicates were done for each genotype, and the RNA samples were used for 3′-end cDNA library construction (QuantSeq 3′ mRNA-seq Library Prep Kit, Lexogen) and high-throughput sequencing (NextSeq 500, Illumina) in which 15–20 M 50 bp, single-end reads were collected for each sample.

Sequence analysis was performed on the Galaxy platform^17^. The reads were mapped to the Drosophila genome (dm6) using HISAT2^18^, expression counts for each gene were tallied by htseq-count^19^, gene expression values were calculated by Cufflinks^20^, and differential gene expression was determined using DESeq2 with the FDR cutoff set at 0.05^21^.

## Results

### The unique C-terminus of Phf7 is necessary to drive masculinization of female germline

*Phf7* is comprised of three zinc fingers classified as PHD domains at the N-terminus and this region makes up about 60% of the length of the protein. The remaining 40% in the C-terminal portion of Phf7 is mysterious in structure and function. This C-terminus of Phf7 shares no significant homology with any known domains, in and outside of the *Drosophila* genus, and prediction programs did not suggest specific structures or domains that may be formed in this region. Previous results have indicated that the Phf7 C-terminus is not always required for all functions of the protein. Male flies lacking *Phf7* exhibit reduced fecundity and this defect can be rescued by a *Phf7* gene without its C-terminus^10^. Such results indicated that Phf7 can regulate spermatogenesis without this part of the protein, and prompted us to ponder if this part of the protein would have any function in the other role Phf7 is known to play: establishment of the male germline sex.

**Figure 1:**
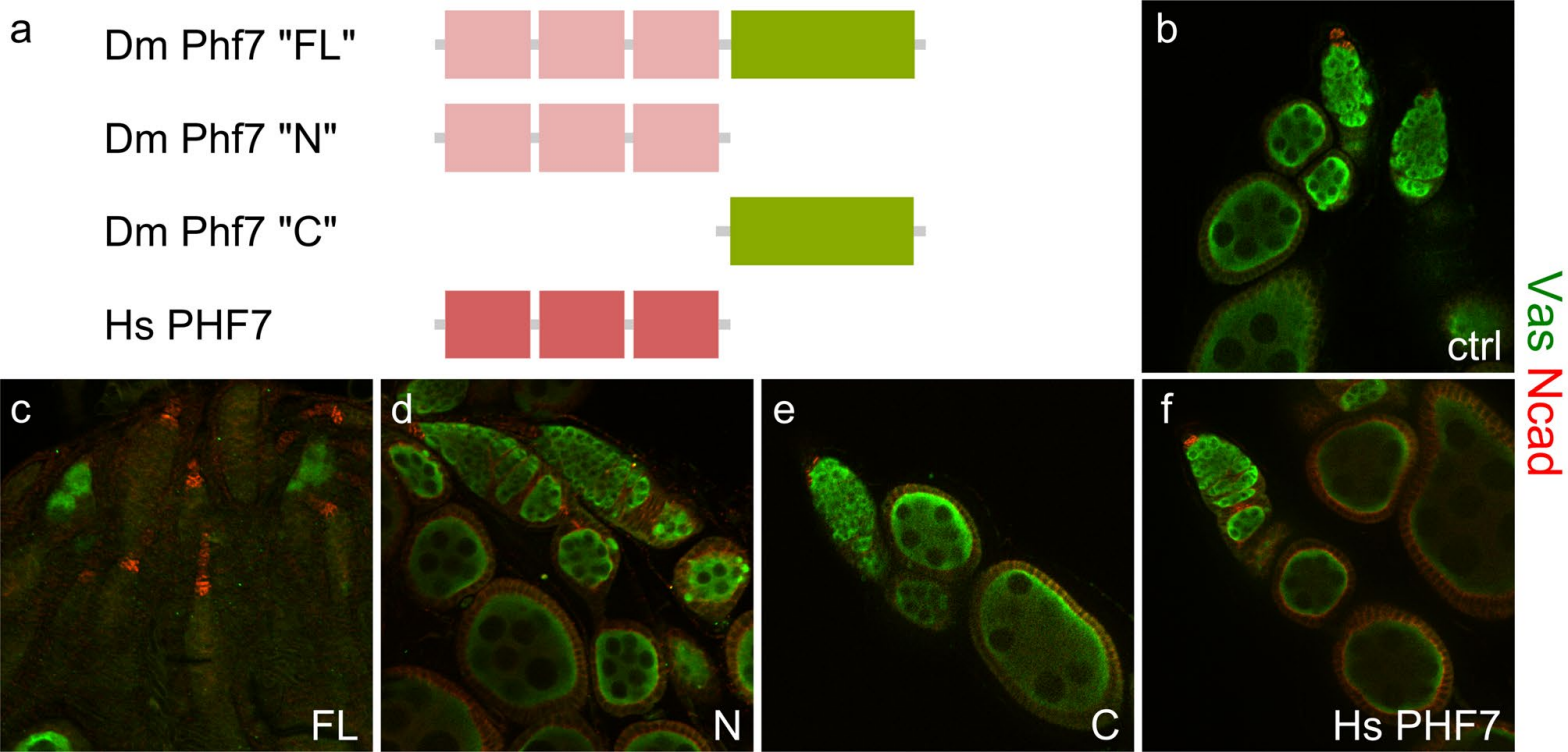
The C-terminus of *D. melanogaster* Phf7 is necessary to cause female germline loss. (**a**), Schematic diagrams of different constructs used in this experiment. “FL” is full-length Phf7 protein, “N” denotes the fruit fly protein without the C-terminus, “C” indicates just the C-terminus alone. Dm is *D. melanogaster* and Hs is short for *H. sapiens*. (**b–f**), Ovaries with germline expression of various forms of Phf7. Vasa is in green, N-cadherin is in red. Genotypes for the panels are: (**b**), *nos-Gal4/* + , (**c**), *nos-Gal4/UAS-Phf7.FL*, (**d**), *nos-Gal4/UAS-Phf7.N*, (**e**), *nos-Gal4/UAS-Phf7.C*, (**f**), *nos-Gal4/UAS-hPhf7.*

To test if the Phf7 C-terminus is involved in male germline sex determination, different *Phf7* fragments were overexpressed in female germ cells using the germline-specific *nanos-Gal4 (nos-Gal4)* to express UAS-driven *Phf7* transgenes. Overexpression of full-length *D. melanogaster Phf7* gene results in a clear loss of germ cell phenotype (Fig. 1a–c), and this is presumed to be due to masculinization of the germ cells, thereby making them sexually incompatible with the surrounding female somatic gonad^7^. This assay was extended to test whether *Phf7* without the C-terminus or the C-terminus alone could also trigger the same effects in the female germline (Fig. 1d,e). Intriguingly, neither caused any female germline phenotypes, suggesting that the entirety of the *Phf7* gene is necessary for this effect. Human PHF7 also lacks this portion of protein and does not cause female germline loss when ectopically expressed (Fig. 1f).

**Figure 2:**
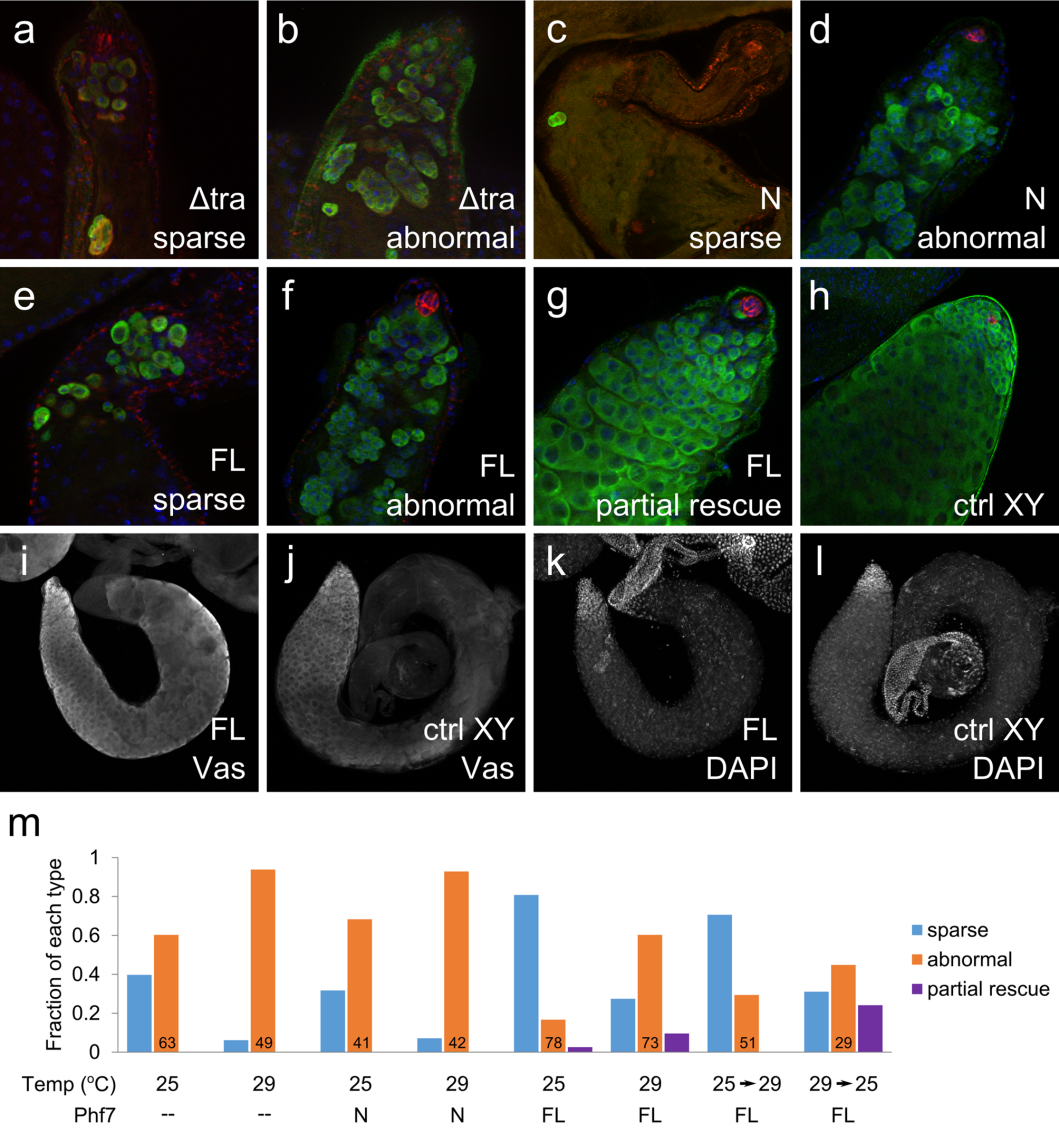
Stimulation of male-like development in female germ cells growing in XX pseudotestes by Phf7. (**a,b**), Δ*tra/tra*^*1*^, *nos-Gal4* pseudotestes. (**a**), sparse type, (**b**), abnormal type. (**c,d**), *UAS-Phf7.N,* Δ*tra/tra*^*1*^, *nos-Gal4* pseudotestes. (**c**), sparse type, (**d**), abnormal type. E–G, *UAS-Phf7.FL,* Δ*tra/tra*^*1*^, *nos-Gal4* pseudotestes. (**e**), sparse type, (**f**), abnormal type, (**g**), partial rescue type. (**h**), *tra*^*1*^, *nos-Gal4/* + testis as a normal control. (**a–h**), Vasa in green, N-cadherin in red. (**i–l)**, comparison between “partial rescue” pseudotestes and normal testes. (i**,j**) show Vasa staining, (**k–l**) show the DAPI signals. (**m**), distribution of different types of pseudotestes under various conditions. Blue, orange, and purple bars indicate the sparse, abnormal, and partial rescue categories respectively. Sample sizes are indicate for each genotype on the graph. The temperatures at which each set of data was performed under as well as the *Phf7* construct expressed are indicated below the graph. N is *UAS-Phf7.N* and FL is *UAS-Phf7.FL.*

Next we wanted to ascertain if the female germline loss caused by overexpression of *Phf7* is indeed due to masculinization of such XX germ cells. This was done by letting these XX germ cells develop in a male somatic environment by manipulating the somatic sex determination genes. Briefly, we utilize XX flies mutant for *transformer (tra, tra*^*1*^*/Df(3L)st-j7)* whose soma would be masculinized whereas the germ cells would be XX and overexpressing various fragments of *Phf7* by a germline driver (*nos-Gal4, UAS-Phf7*). It is well documented that XX germ cells developing in a male soma cannot survive and differentiate properly^2,22,23^, and there are two typical phenotypes of these XX germ cells. They are either almost all lost in the adult pseudotestes, or substantial germ cells remain albeit in a relatively undifferentiated state in which the germ cells are small and lack the characteristics of later-stage development (Fig. 2a,b). In this paper we name these two types as “sparse” and “abnormal”, respectively, and they each make up about half of the pseudotestes we have examined in the absence of other genetic manipulations (Fig. 2m).

Overexpression of *Phf7* changes the distribution of these phenotypes of the XX germ cells. When the N-terminus alone construct is expressed, most of the gonads contain significant numbers of small germ cells that fail to progress (“abnormal”, Fig. 2c,d,m). When full-length *Phf7* is expressed, most pseudotestes contain minimal numbers of germ cells (“sparse”, Fig. 2e), but about 10% contain germ cells that mirror normal spermatogenesis at least up to the late spermatocyte stage (“partial rescue”, Fig. 2g–m). This partial rescue requires higher levels of Phf7 expression which is done with flies being grown at 29 °C to enhance the activity of GAL4 to drive transgene expression; growing them at 25 °C is insufficient to initiate this level of male germline rescue (Fig. 2m). Interestingly, there was also a higher ratio of pseudotestes that contained small, undifferentiated germline (“abnormal”, Fig. 2f,m) at 29 °C compared to when flies were grown at 25 °C.

The temperature-dependent germline masculinization by *Phf7* allowed us to further address the developmental time point at which this process occurs. This was tested by raising the flies for 5 days at 25 °C and the remaining 5 days at 29 °C, or the reverse. We find that pseudotestes containing germline that underwent substantial differentiation (“partial rescue”) were only observed when flies were grown first at 29 °C and then 25 °C (Fig. 2m). The pseudotestes phenotype of flies grown initially at 25 °C and then 29 °C were very similar to those continuously maintained at 25 °C. All the results from these *Phf7-*expressing experiments indicate three things. First, the period most critical for choosing the male germline fate is early in development, in line with observations from other studies that showed the earliest signs of sexual dimorphism in the germline begins during embryogenesis^5,6^. Second, expressing *Phf7* highly later in development is not sufficient to overcome the defects in determining the proper sexual fate earlier. Lastly, as the female germline loss assay correctly suggested, the masculinizing effect of Phf7 in the germline requires its C-terminus.

### The unique C-terminus of Phf7 is necessary for sex-specific gene expression

The partial penetrance of the spermatogenesis rescue phenotype induced by full length *Phf7* in female germ cells developing in male somatic environments prompted us to investigate possible differences in the pseudotestes that exhibit different grades of germline development. Specifically, expression of several genes known to have sex-dependent expression patterns in the germ cells were examined.

**Figure 3:**
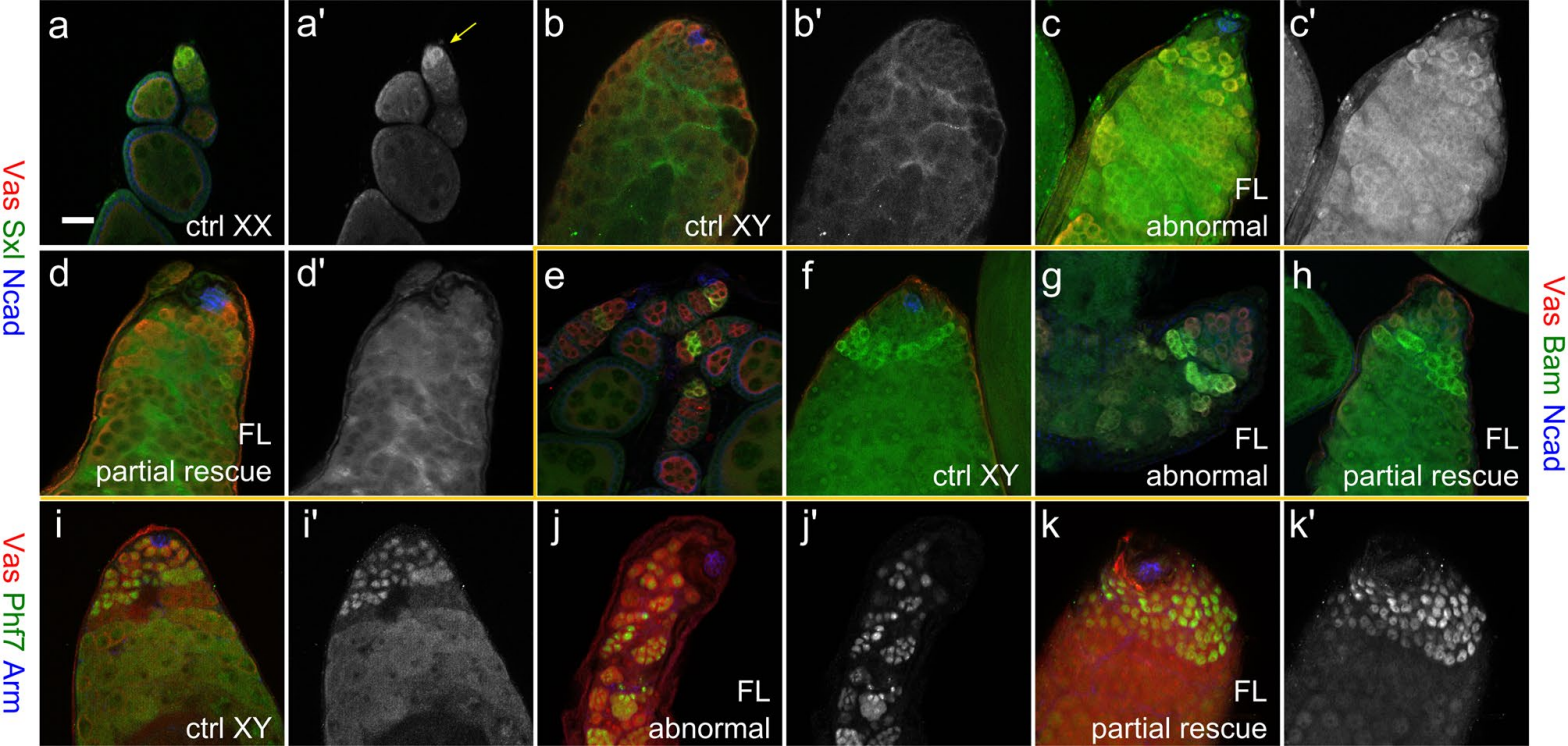
Expression of sex-specific markers in the pseudotestes. (**a**–**d**), Sxl staining in wild-type ovaries (**a**), wild-type testis (**b**), and abnormal (**c**) and partial rescue (**d**) type pseudotestes of *UAS-Phf7.FL,* Δ*tra/tra*^*1*^, *nos-Gal4*. Vasa is in red, Sxl in green, and N-cadherin in blue. (**a**′**–d**′) display just the Sxl signals alone. The yellow arrow in A′ indicate the germline stem cells in an ovariole that stain Sxl clearly. (**e–h)**, Staining of Bam in wild-type ovary (**e**), wild-type testis (**f**), and abnormal (**g**) and partial rescue (**h**) type pseudotestes of *UAS-Phf7.FL,* Δ*tra/tra*^*1*^, *nos-Gal4*. Vasa is in red, Bam in green, and N-cadherin in blue. (**i–k)**, Phf7 expression in wild-type testes (**i**), and abnormal (**j**) and partial rescue (**k**) type pseudotestes of *UAS-Phf7.FL,* Δ*tra/tra*^*1*^, *nos-Gal4*. Vasa is in red, Phf7 in green, and Armadillo in blue. (**i**′**–k**′) display the Phf7 channel alone.

Sex lethal (Sxl) is involved in the sexual development of both the female germline and soma^6,24^, and its expression is the highest in germline stem cells and cystoblasts in the ovary while undetectable in normal testes^25,26^ (Fig. 3a,b). Intriguingly, in the “abnormal” category of pseudotestis, most germ cells clearly express Sxl cytoplasmically regardless of whether they over-express *Phf7*, or which form thereof (Fig. 3c, Supplementary Fig. S1a,b). The “sparse” type pseudotestes were difficult to analyze due to the very small numbers of remaining germ cells. In contrast, germ cells in the “partial rescue” category do not express Sxl (Fig. 3d). These observations indicate that germ cells in the “abnormal” category are not able to fully suppress the female germline program.

We next looked at expression of Bam, a pro-differentiation factor in both male and female germline that is expressed in slightly different stages: in males it is mainly in the 4–8 cell spermatogonia and in females 2–4 cell oogonia^27,28^ (Fig. 3e–f). The BAM expression patterns in all pseudotestes examined, regardless of phenotype and genotype, were quite similar and we find the protein to be present largely in the 4–8 cell stage (Fig. 3e–h, Supplementary Fig. 1e,f), in line with what is observed in normal testes.

Phf7 is yet another protein exhibiting sex-biased expression: it is substantially expressed in the nuclei of male germline stem cells and spermatogonia up to the 8-cell stage but undetectable in the female germline by immunostaining^9^. The fact that the experiments here involve overexpression of *Phf7* complicates the analysis of Phf7 staining results. However, as the epitope of the Phf7 antibody is in the C-terminus, it is still meaningful to examine Phf7 expression in pseudotestes without *Phf7* overexpression as well as those expressing just the N-terminus of Phf7 as we wondered whether Phf7 expression from the endogenous locus would be different in XX germ cells exhibiting different degrees of male-like development. We found that Phf7 is expressed in all germ cells of the “abnormal” type as well as those in “partial rescue” that resemble germline stem cells and spermatogonia (Fig. 3i–k, Supplementary Fig. 1c,d). Surprisingly, we did not see higher expression in germ cells that overexpress the full-length *Phf7* construct, even if they are of the “partial rescue” category.

These results, taken together with the Bam staining results, indicate that most XX germ cells developing in the male gonad take on a partial male identity, possibly with help from signals from the male soma. Nonetheless, without the coordination of germline-intrinsic mechanisms, the male germline program cannot be fully installed. Phf7, with its C-terminus, is able to provide that critical germline-intrinsic information to trigger the proper male program.

### Phf7 up-regulates expression of most of its downstream genes in the embryonic germline

To understand how *Phf7* initiates the male germline program, we wanted to examine the genes regulated by *Phf7* to reveal the factors that are important in turning on the male germline program. We have previously performed a related experiment of looking for *Phf7* target genes in the adult testis of *bam* mutants which are enriched for spermatogonia that express *Phf7* highly^9^. However, our latest results highlight the importance of addressing this question in a stage-specific manner as the role of Phf7 in male germline development changes over time, and the genes that Phf7 regulates likely differ for these distinct roles.

**Figure 4:**
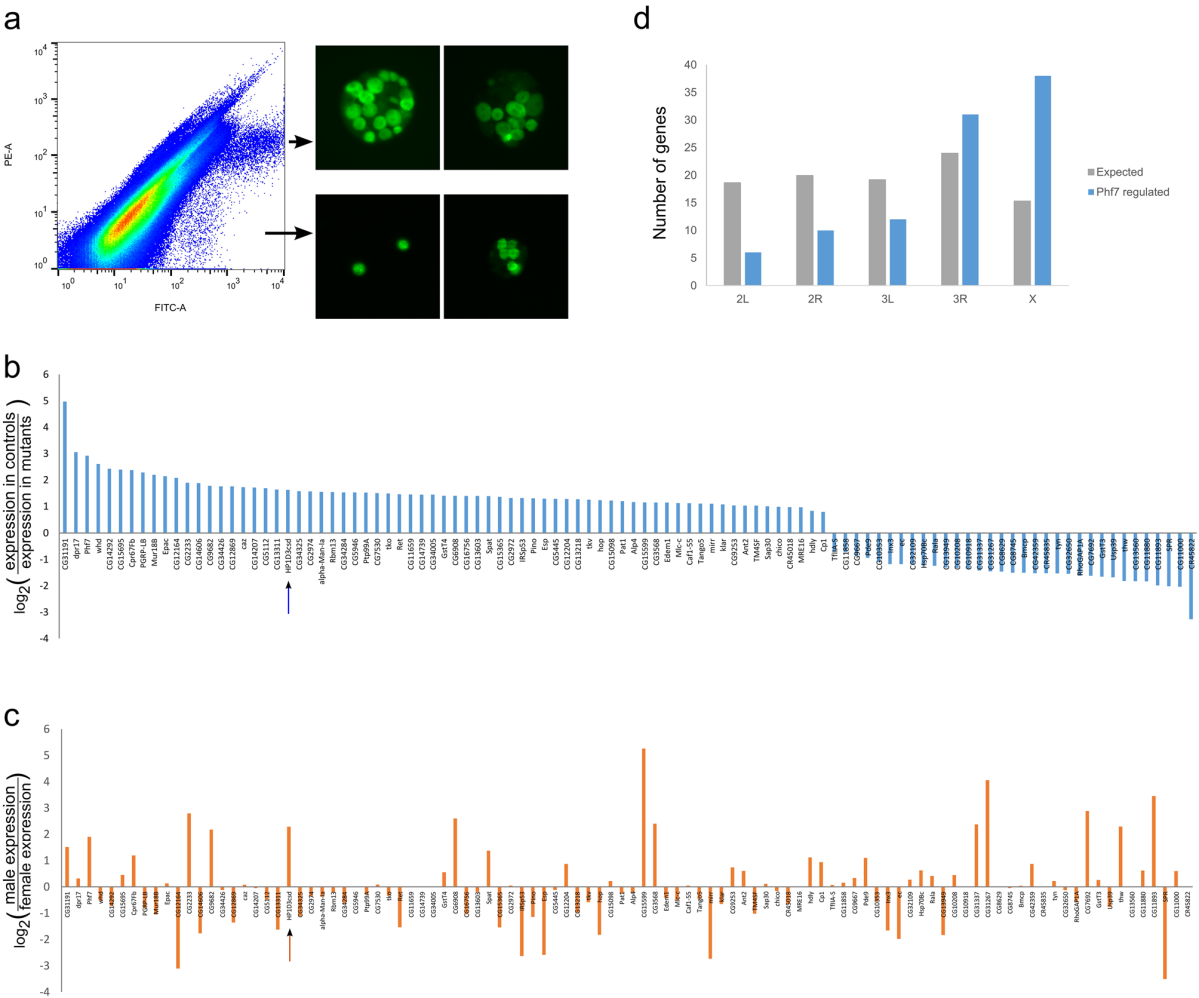
Transcriptome profiling of embryonic gonads with or without *Phf7*. (**a**), FACS plot of homogenate from *vas-GFP* late embryos. The GFP + particles are distributed in two patches, and representative images of gonads from each population are shown to the right of the FACS plot. Gonads from the upper population are more intact and were collected for RNA-seq. (**b)**, Genes whose expression are significantly changed by the *Phf7* mutation are plotted. The *y*-axis is the log-2 value of the expression ratio of control to mutant. *HP1D3csd* is indicated with an arrow. (**c**), Sex-expression ratio of *Phf7-*regulated genes in *bam-*mutant adult testis versus ovary based on another study^29^. *HP1D3csd* is indicated with an arrow. (**d**), Distribution of *Phf7-*regulated genes across different major fruit fly chromosomes. Gray bars are the expected number of genes on that chromosome based on their relative numbers of genes; blue bars represent distribution of *Phf7-*regulated embryonic targets. The observed distribution is significantly different from the expected one based on chi-squared analysis (*P* < 0.001).

To identify the genes *Phf7* regulates in the embryonic germline, we FACS-sorted control or *Phf7-*deficient late embryonic gonads that carry the germline-specific *vas-GFP* transgene. Gonads from 18 to 22 h embryos were obtained by mechanical disruption to dislodge the gonads from the surrounding tissues, and the gonads mostly stay as a whole though some germ cells are released from the gonads in this process (Fig. 4a). The homogenates were subsequently passed through a FACS sorter so that we can separate out GFP + particles, whose distribution on the FACS plot turned out to be rather broad (Fig. 4a). Examination of the morphology of GFP + particles from different parts of the FACS plot under a fluorescence microscope confirmed that the sorted particles are enriched for more intact gonads. We opted against further treatment of the gonads with enzymes to obtain single germ cells as we wanted to reduce disturbance on the germ cell transcriptome. This means that the sample contains both germ cells and somatic cells, but we reasoned that the contribution of germ cells to the total RNA purified from gonads is substantial enough that we could detect expression changes resulting from the absence of *Phf7*. The samples we collected included both male and female samples; male gonads are bigger than female gonads in late embryogenesis, but we found out that this difference was not sufficient for effective separation by FACS. The impact of this impurity is likely also limited as female germ cells are quiescent in this period and our samples would contain more male germ cells than female germ cells. Previous experiments looking for gene expression differences between male and female germline have also resulted mostly in those with higher expression levels in the male embryonic germline^4^.

Two biological replicates for both control and *Phf7-*mutant gonads were processed for transcriptome analysis by next generation sequencing. The correlation coefficients between the replicates were good: 0.91547 for the control datasets and 0.94868 for the mutant datasets. Next we examined whether expression of genes known to be present in the germline or gonad of early stage 17 embryos is indeed observed and at comparable levels in all samples. We observed clear and consistent expression for germ cell genes such as *nos, vasa,* and *piwi,* whereas genes present in earlier pole cells like *pgc* or are turned on after differentiation has started such as *bam* are expressed minimally (Supplementary Fig. 2a). We also looked at genes reported to exhibit sex-enriched expression patterns at this stage of gonads and found most of them to be expressed (Supplementary Fig. 2b,c)^4^, indicating that our preparations contained a mix of embryonic testes and ovaries.

We found 97 genes to show significantly different expression levels between control and *Phf7-*deficient samples (Fig. 4b). Of those, expression levels of roughly two-thirds were lower in *Phf7-*mutants indicating that *Phf7* would normally stimulate their expression. Intriguingly, this is opposite of what we found of genes regulated by *Phf7* in the adult germline^9^. We will note that this analyses does not identify whether these genes are directly targeted by Phf7 or whether their expression changes are secondary to the immediate effects of Phf7. We examined whether the genes regulated by *Phf7* in the embryonic germline exhibit sex-biased expression and found that about they are evenly split between being male- and female-biased in undifferentiated adult germline (Fig. 4c). There are currently no comprehensive gene expression databases for the male versus female embryonic germline, thus we do not know if the *Phf7-*regulated genes exhibit a stronger male-biased trend during embryogenesis. Curiously, there is minimal overlap between the genes regulated in the embryonic and adult germline by *Phf7* (Supplementary Fig. 2d), though we do note that all of the ones that overlap are male-biased. These differences in downstream genes likely reflect the differing roles of Phf7 in the embryonic and adult germline. Interestingly, the genes regulated by *Phf7* in embryogenesis are enriched for being on the X chromosome (Fig. 4d). When we re-examined the chromosomal distribution of those regulated by *Phf7* in adults, we found a similar trend (Supplementary Fig. 2e), suggesting that X chromosome genes could be preferentially targeted by Phf7.

As Phf7 appears to regulate gene expression differently in the embryonic and adult germline, and the Phf7 C-terminus appears to act mainly in germline masculinization, a process that occurs normally during embryogenesis, we wondered whether the C-terminal portion of Phf7 may function like a transcriptional activating domain to enhance target gene expression. This was tested by replacing the activation domain of GAL4 with the Phf7 C-terminus to create a fusion protein between the DNA binding domain of GAL4 with the C-terminus of Phf7. A construct containing this fusion protein placed under the regulation of the ubiquitin promoter was co-transfected with *UAS-GFP* into S2 cells to test the ability of the Phf7 C-terminus to activate expression of GFP. However, we did not observe GFP expression in such S2 cells whereas the positive control treatment (*Ubi-GAL4, UAS-GFP*) resulted in a clear population of GFP + cells (Supplementary Fig. 3a,b). Thus this part of the Phf7 protein does not function as a canonical transcriptional activation domain in S2 cells.

### HP1D3csd acts downstream of Phf7 to induce germline masculinization

**Figure 5:**
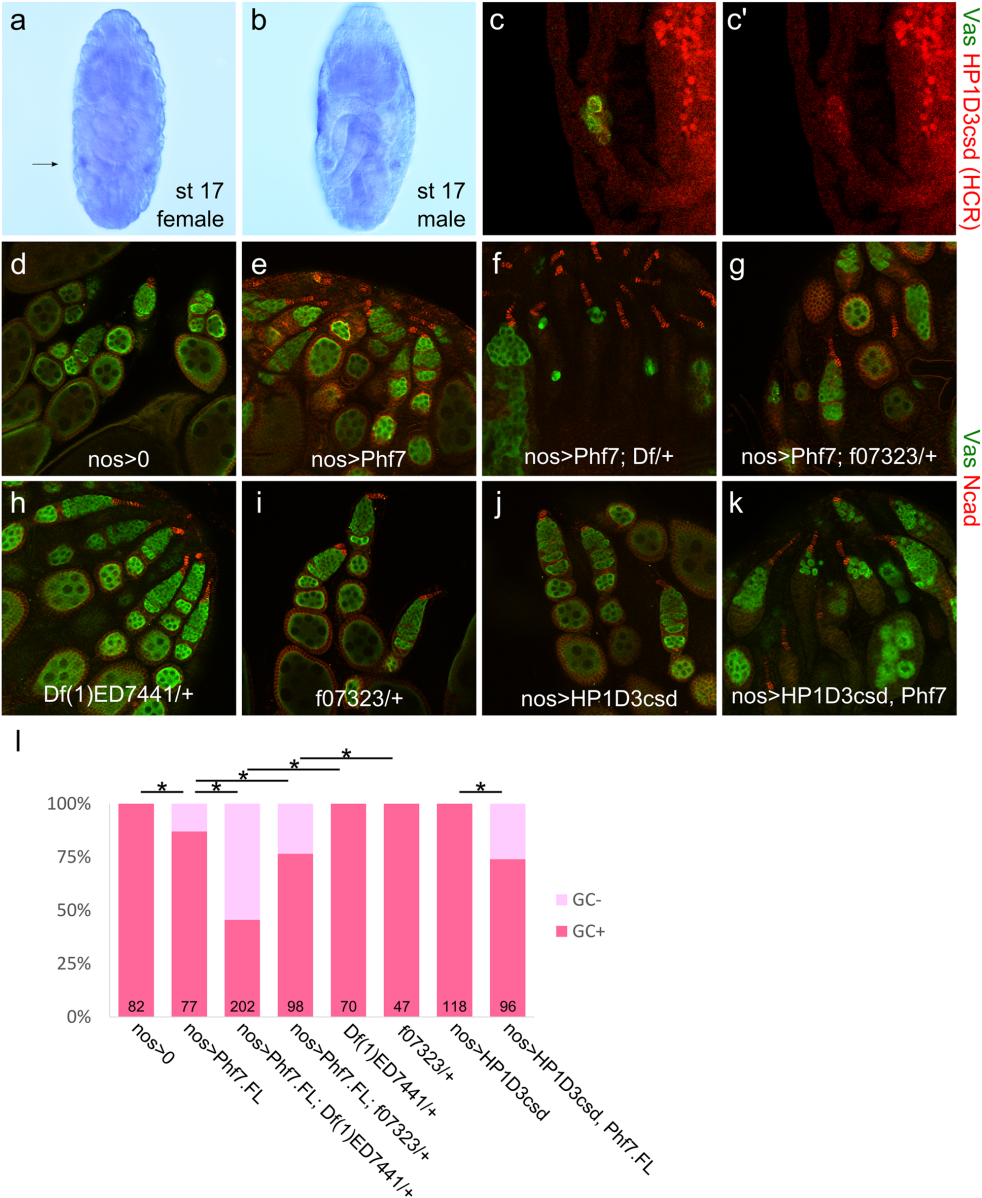
Genetic interaction between *Phf7* and *HP1D3csd* on the female germline loss caused by *Phf7* overexpression. (**a**,**b**), in situ hybridization of stage 17 embryos with an *HP1D3csd* probe. (**a**), Female embryo; arrow indicates position of gonad on one side. (**b**), Male embryo. (**c**), HCR staining of *HP1D3csd* transcripts in an unsexed stage 17 embryo. Colocalization with the Vas (green) signal indicate the *HP1D3csd* signals are in germ cells. (**c**'), HCR signal alone. (**d–k**), Ovaries of various genotypes stained with Vasa (green) and N-cadherin (red). (**d**), *nos-Gal4/* + *,* (**e**), *nos-Gal4/UAS-Phf7.FL*, (**f**), *Df(1)ED7441/* + *; nos-Gal4/UAS-Phf7.FL*, (**g**), *HP1D3csd*^*f07323*^*/* + *; nos-Gal4/UAS-Phf7.FL*, (**h**), *Df(1)ED7441/* + , (**i**), *HP1D3csd*^*f07323*^*/* + , (**j**), *nos-Gal4/UAS-HP1D3csd.HA*, (**k**), *nos-Gal4/UAS-HP1D3csd.HA, UAS-Phf7.FL*. (**l**), Quantitation of the fraction of ovarioles that contain no germline in different genotypes. Sample sizes and genotypes are indicated on the bottom of the bars. Dark and light pink portions of the bars denote the fraction of ovaries with and without germline. * indicates *P* < 0.05 with chi-square tests.

One of the candidate genes regulated by *Phf7* in embryogenesis is the X-encoded *HP1D3csd.* This gene encodes a small, ~ 20 kD protein predicted to resemble chromoshadow domains that are typically found in HP1 proteins and mediate dimerization and interactions with other chromatin factors^12,30–32^. As Phf7 is a chromatin-associated protein, we became intrigued by the possibility that HP1D3csd may assist Phf7 in regulating expression of genes important for setting up the male germline sex. *HP1D3csd* is expressed germline-specifically in the embryo (Fig. 5a–c). Its transcripts are present in germline of both sexes but become male-biased later (Figs. 4c, 5a,b).

To test for possible interactions between *Phf7* and *HP1D3csd*, we asked if *HP1D3csd* could modify the loss of female germline caused by ectopic expression of *Phf7*. We first addressed what would happen with a lower dose of *HP1D3csd* by using two mutants of *HP1D3csd*, a transposon-insertion in the 3′ end of the coding sequence of *HP1D3csd* which is possibly a hypomorphic allele (*HP1D3csd*^*f07323*^), and a deficiency covering *HP1D3csd* (*Df(1)ED7441*)*.* When one copy of *HP1D3csd* was mutated (*HP1D3csd*^*f07323*^*/* + or *Df(1)ED7441/* +), the extent of female germline loss caused by *Phf7* was exacerbated compared to when *HP1D3csd* is intact (Fig. 5d–g,l). Ovaries heterozygous for *HP1D3csd*^*f07323*^ or *Df(1)ED7441* alone did not show any germline defects (Fig. 5h,i,l), indicating that the phenotype enhancement is due to a genetic interaction between *Phf7* and *HP1D3csd.*

The relationship between *Phf7* and *HP1D3csd* was further investigated by overexpressing *HP1D3csd* along with *Phf7* in female germ cells. We used an HA-tagged transgene driven by UAS (*UAS-HP1D3csd.HA*) from the FlyORF collection. Quite surprisingly, overexpressing the two genes together also causes more severe germline loss than when just *Phf7* is overexpressed (Fig. 5j–l). It should be noted that when *HP1D3csd* and *Phf7* are co-expressed using *nos-Gal4*, expression levels of each of the genes are expected to drop, which should alleviate the germline loss effect induced by *Phf7* overexpression. The fact that the opposite was observed indicates that overexpressing *HP1D3csd* clearly leads to a stronger germline loss phenotype. These results suggest that HP1D3csd is functionally associated with Phf7, but as both reduced and increased doses of HP1D3csd enhanced the female germline loss phenotype mediated by Phf7, the exact relationship between Phf7 and HP1D3csd is unclear.

**Figure 6:**
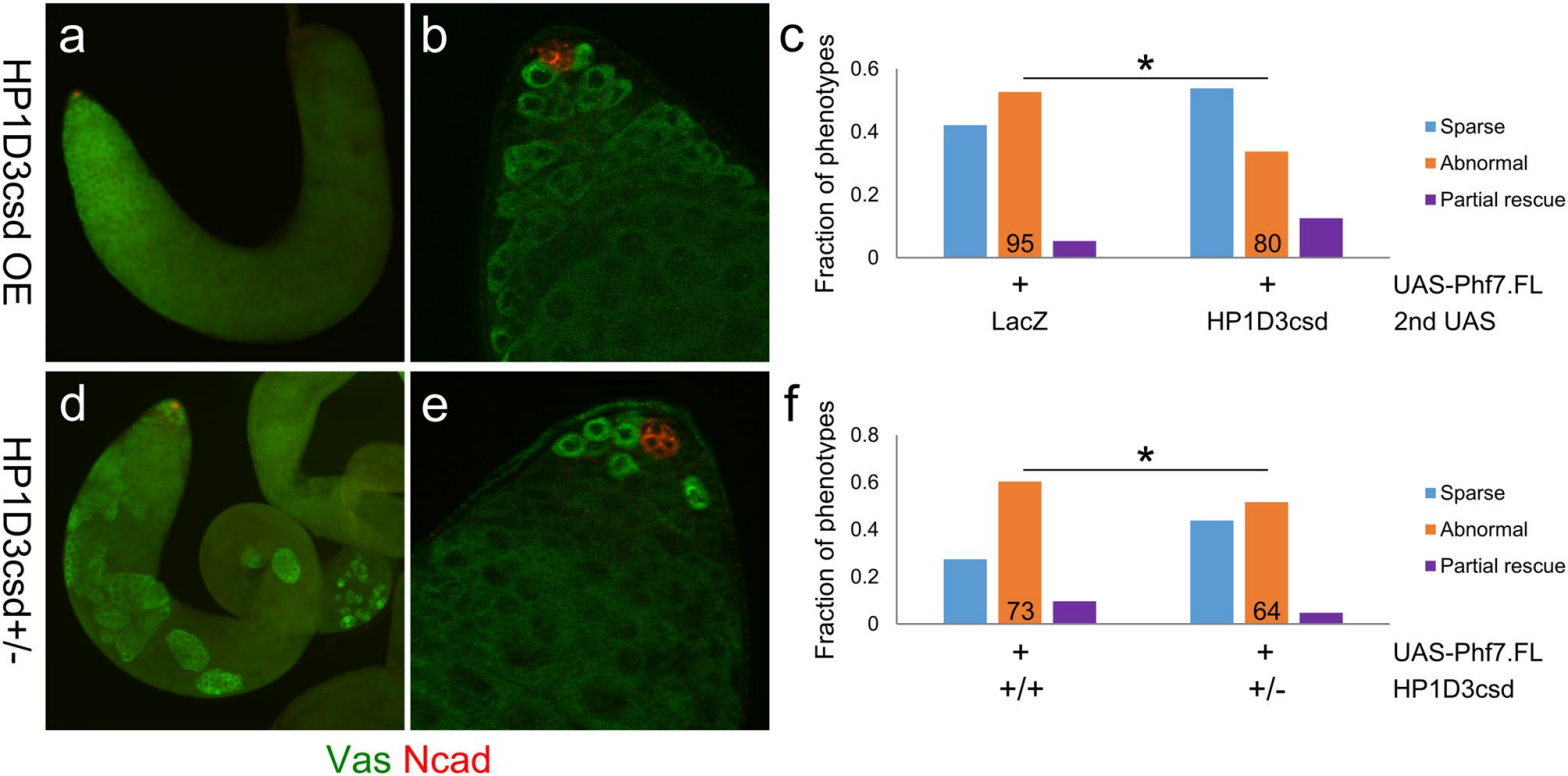
HP1D3csd affects the ability of Phf7 to induce germline masculinization in a dose-dependent manner. (**a**,**b**), Example of a pseudotestis with its XX germ cells overexpressing both *Phf7* and *HP1D3csd* (*UAS-Phf7.FL, UAS-HP1D3csd.HA,* Δ*tra/tra*^*1*^, *nos-Gal4*) that exhibited “partial rescue” of spermatogenesis. Vas is in green and N-cad is in red. (**a**), View of the entire pseudotestis. (**b**), View of the pseudotestis apical tip. (**c**), Quantitation of the different types of phenotypes observed for pseudotestes with XX germ cells overexpressing *Phf7* and either *HP1D3csd* or a control construct (*LacZ*). The numbers in the graph indicate sample sizes. * indicates *P* < 0.05 with chi-square tests. (**d,e**), A pseudotestis overexpressing *Phf7* but lacking a functional copy of *HP1D3csd* (*HP1D3csd*^*f07323*^*/* + *; UAS-Phf7.FL,* Δ*tra/tra*^*1*^, *nos-Gal4*) exhibiting “partial rescue” of spermatogenesis. Vas is in green and N-cad is in red. (**d**), Image of the entire pseudotestis. (**e**), Image of the pseudotestis apical tip. (**f**), Quantitation of the different types of phenotypes observed for pseudotestes with their XX germ cells overexpressing *Phf7* and either wildtype or harboring a mutant copy of *HP1D3csd*. The numbers in the graph indicate sample sizes. * indicates *P* < 0.05 with chi-square tests.

To clarify the role of HP1D3csd in germline sexual development and how it interacts with Phf7, we carried out a second set of experiments to test for genetic interaction between these two factors: we investigated whether HP1D3csd can modify the ability of Phf7 to induce partial spermatogenesis in female germ cells that develop in a male somatic gonad. These experiments were carried out at 29 °C, the temperature at which we observed partial spermatogenesis to occur in the female germline overexpressing *Phf7* developing in a male soma. When HP1D3csd was overexpressed along with Phf7, we found that the frequencies of pseudotestes that exhibited partial spermatogenesis (“partial rescue” phenotype) was increased compared to samples that expressed an unrelated protein (LacZ) together with Phf7 (Fig. 6c). The extent of spermatogenesis that occurred in these samples is comparable to what is achieved with just Phf7 overexpression (Figs. 6a,b vs. 2g,i,k). The fraction of partial rescue type in samples that overexpress both Phf7 and LacZ is lower than that of pseudotestes that only overexpress Phf7 (Fig. 6f); this is most likely because expression of Phf7 is reduced in the former genotype with both Phf7 and LacZ being driven by a single *nos-Gal4* driver.

We also performed the reverse experiment by examining pseudotestes that overexpress *Phf7* and are heterozygous for the *f07323* allele of *HP1D3csd*. The rate of such pseudotestes that show partial spermatogenesis was lower than what we observed for samples that overexpressed *Phf7* but were wild-type for *HP1D3csd* (Fig. 6f). In addition, the extent of spermatogenesis rescue in most of the pseudotestes that have a mutant copy of *HP1D3csd* was more limited than most of those in the same phenotypic category for other genotypes (Figs. 6c,d vs. a,b, 2g,i). These results show that a single *HP1D3csd* mutant copy diminished the ability of Phf7 to trigger male germline development. Taken together, results of our experiments clearly demonstrate that *Phf7* and *HP1D3csd* interact genetically. Furthermore, they indicate that HP1D3csd acts downstream of Phf7 to facilitate germline masculinization.

## Discussion

In this study we investigated how Phf7 regulates sex determination in the embryonic germline, and one of our interesting finding is that the unusual C-terminus of Phf7 is necessary for its effects in germline masculinization. The N-terminus of Phf7 is a conserved module comprised of three zinc fingers, of which at least one is functionally essential^33^, and this part of the Phf7 protein evolved from G2E3 (G2/M E3 Ubiquitin Ligase), a protein also made up of three zinc fingers^10^. In contrast, the C-terminus of Phf7 is evolutionarily novel and is not similar to any known domains, suggesting that this domain is undergoing very rapid evolution, a feature not uncommon for factors involved in sex determination^34,35^.

We previously conducted a phylogenetic analysis of Phf7 proteins across the species tree, and surprisingly found that Phf7 in insects and amniotes do not share a common ancestor^10^. Those findings with our latest results indicate that Phf7 in these two animal branches are not orthologous to each other, and that the emergence of the novel C-terminus is likely a unique event that occurred in *Drosophila* to regulate sexual differentiation in the germline. Recently, mouse Phf7 was demonstrated to be expressed in spermatocytes and can ubiquitinate histones to facilitate histone to protamine exchange^8,36^. These show that the expression patterns and functions of *D. melanogaster* and mouse Phf7 are different, albeit both acting on the male germline. These observations further suggest that the C-terminus of *D. melanogaster* PHF7 evolved onto an existing module of three zinc fingers, thereby creating new ways to regulating germline sexual development. This is a very interesting example that adds to the collection of diverse mechanisms in sex determination.

What does this uncommon C-terminus of PHF7 do? The two most intuitive ideas are that it acts as a transactivation domain like those found in transcription factors, or that it can recruit other effector molecules through protein–protein interactions. The former idea did not hold up when tested in S2 cells. The possibility that the Phf7 C-terminus acts as a bridge between its histone-associating N-terminus and other transcription factors or chromatin factors to alter target gene expression is an appealing one but there is currently no direct data that support this idea.

We further looked for downstream effectors of Phf7 in the embryonic germline, and revealed that HP1D3csd is activated by Phf7 to regulate germline masculinization. We performed two different genetic tests, and while both indicated that Phf7 and HP1D3csd genetically interact, there were some differences in the results. In the *Phf7-*induced female germline loss assay, we found both reduction and gain of HP1D3csd expression exacerbated the Phf7-induced phenotype. In comparison, in the spermatogenesis rescue assay, loss of one *HP1D3csd* copy hampered rescue whereas *HP1D3csd* overexpression enhanced spermatogenesis in XX germ cells. The latter experiment is a more direct assay of germline masculinization whereas germline loss can potentially be caused by secondary effects unrelated to sexual development. Therefore, we think the results of the spermatogenesis rescue experiments more accurately reflect the relationship between Phf7 and HP1D3csd. In addition to our transcriptome results, HP1D3csd has been identified along with Phf7 to be a part of sex-biased mechanisms in other contexts not limited to the germline^37,38^. These also support the model that Phf7 and HP1D3csd function synergistically.

Phf7 regulates male germline development, and it can associate with the active histone mark methylated H3K4^7^, but it is unclear what Phf7 then does to regulate expression of target genetic loci. H3K9 methylation has also been reported to be important for maintaining sexual differentiation programs in the *Drosophila* germline^39^. The identification of HP1D3csd as an important downstream factor provides interesting new ideas regarding how the male germline program is initiated and regulated. CSDs have been shown to interact with various chromatin remodelers, thus one appealing model would be that Phf7 can activate or even recruit HP1D3csd to loci important for germline masculinization. This would in turn bring chromatin remodelers to such genes for expression activation and regulation and initiate male-development of the germline. Given our other finding in this study that the C-terminus of Phf7 is an essential part of this process, it would be very interesting to now study which of these factors interact and cooperate with one another.

## Supplementary Information

Supplementary Information

## Data Availability

The datasets generated during the current study are available at the NCBI Gene Expression Omnibus (GEO; https://www.ncbi.nlm.nih.gov/geo/) under accession number GSE167380.
