# Supplementary material for: Germline masculinization by Phf7 in D. melanogaster requires its evolutionarily novel C-terminus and the HP1-family protein HP1D3csd
Source: Sci Rep. 2021 Mar 18;11:6308. doi: 10.1038/s41598-021-85560-4 (PMC7973481; doi:10.1038/s41598-021-85560-4)
Supplement: Supplementary file 1 — Supplementary Information [file 41598_2021_85560_MOESM1_ESM.pdf]

Germline masculinization by *Phf7* in *D. melanogaster* requires its evolutionarily novel C-terminus and the *HP1*-family protein HP1D3csd

Shu Yuan Yang

Supplementary Figures

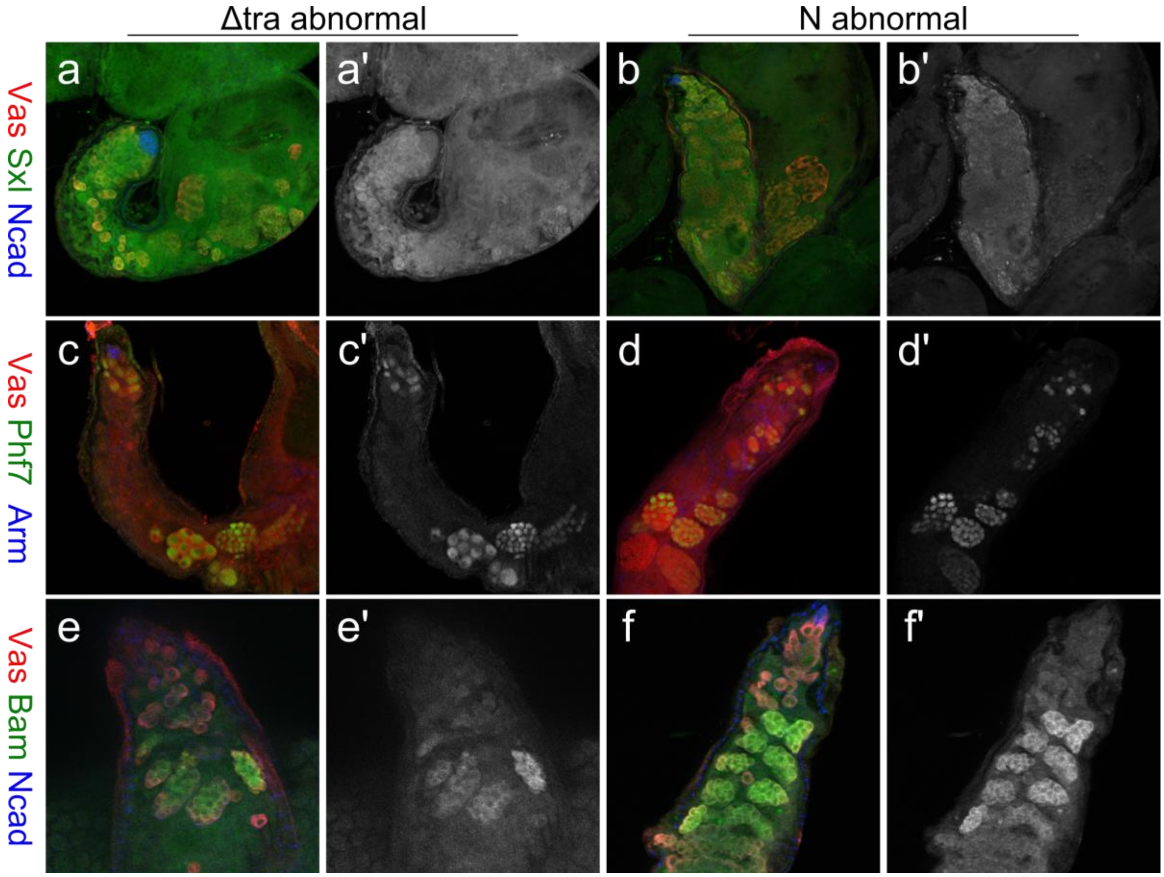

Supplementary Figure 1. Expression analysis of sex-specific factors in pseudotestes. a-**b**, Sxl staining (green) together with Vasa (red) and N-cadherin (blue). **c**-**d**, Phf7 staining (green) along with Vasa (red) and N-cadherin (blue). **e**-**f**, Bam staining (green) in addition to Vasa (red) and N-cadherin (blue). **a**, **c**, **e**, *Δtra/tra¹*, *nos-Gal4*, **b**, **d**, **f**, *UAS-Phf7.N, Δtra/tra¹*, *nos-Gal4*. **a**′-**b**′, **c**′-**d**′, and **e**′-**f**′ depict signals from the green channels (Sxl, Phf7, and Bam, respectively) alone.

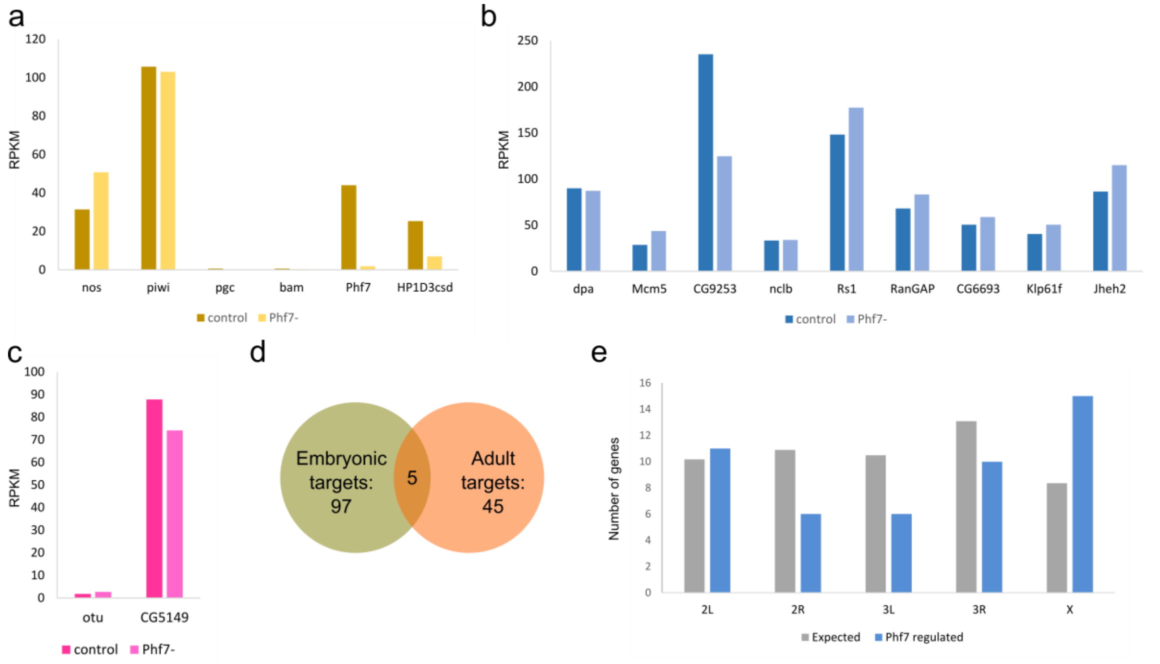

Supplementary Figure 2. Summary of RNA-seq results. a, Expression of various germline genes in the control (dark yellow) and *Phf7*-mutant (light yellow) embryonic gonads. **b**, Expression levels of genes enriched in the male late embryonic gonad in control (dark blue) and *Phf7*-mutant (light blue) samples. **c**, Expression levels of genes enriched in the female late embryonic gonad of control (dark pink) and *Phf7*-mutant (light pink) genotypes. **d**, Venn diagram depicting the overlap between *Phf7*-regulated genes in the embryonic vs. adult germline. **e**, Chromosomal distribution of the adult *Phf7* target dataset from a previous study[9]. Gray bars indicate the expected number of genes on each chromosome in the absence of any bias. Blue bars designate the observed distribution.

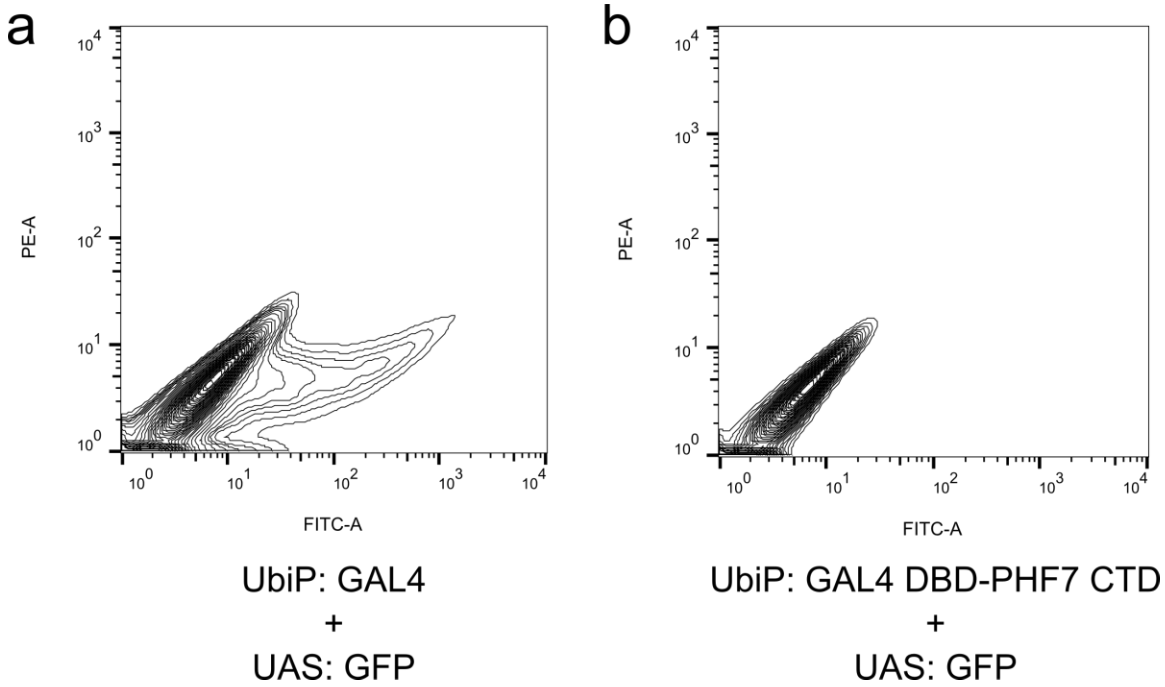

Supplementary Figure 3. FACS profiles of S2 cells transfected with different reporter constructs after 48 hours. a, S2 cells transfected with *UbiP-Gal4* and *UAS-GFP* show a clear GFP+ cell population. **b**, S2 cells transfected with *UbiP-Gal4 DBD/PHF7.C* fusion construct together with *UAS-GFP* cannot promote GFP expression.
